# Rural-urban differences in the clinico-pathologic profiles of Jamaican men with prostate cancer

**DOI:** 10.1186/s13027-015-0023-z

**Published:** 2015-09-28

**Authors:** William D. Aiken, Kieron S. Jones, Camille Ragin, Kenneth James

**Affiliations:** Division of Urology, Section of Surgery, The Department of Surgery, Radiology, Anaesthesia & Intensive care, Faculty of Medical Sciences, University of the West Indies, Kingston 7, Mona, W.I. Jamaica; Hargreaves Memorial Hospital, 32 Hargreaves Avenue Mandeville, Manchester, W.I. Jamaica; The Fox Chase Cancer Center, 333 Cottman Avenue, Philadelphia, PA 19111 USA; Section of Community Health, The Department of Community Health and Psychiatry, Faculty of Medical Sciences, University of the West Indies, Kingston 7, Mona, W.I. Jamaica

**Keywords:** Prostate cancer, Black men, African American, Rural population, Urban population, Health services accessibility, Awareness, Early detection of cancer, Health disparities

## Abstract

**Background:**

Prostate cancer causes the highest number of cancer-related deaths in Jamaican men. It is not known whether rural-dwelling men present with worse disease than urban-dwelling men at initial presentation. Since rural and urban-dwelling Jamaicans are predominantly of African descent and generally similar in respect of racial composition, if any significant variation in initial presentation were found, it would suggest that these are likely due to differences in awareness, access to care, and screening patterns.

**Methods:**

The medical records of rural and urban-dwelling patients with prostate cancer were compared. Patients’ age at presentation, initial prostate-specific antigen level, digital rectal examination findings, biopsy Gleason scores and initial treatment received were compared using bivariate and logistic regression analyses.

**Results:**

In unadjusted analyses rural-dwelling men were older compared to urban-dwelling men (72 years versus 68.5 years, *p* = 0.035), had higher median PSA values (22.9 ng/ml versus 18 ng/ml, *p* = 0.001), higher local tumour stage (65.2 % versus 34.8 % T3 disease; 73.7 % versus 26.3 % T4 disease; *p* = 0.005), higher mean Gleason scores (*p* = 0.048) and more non-curative initial treatments. Local tumour stage was the only statistically significant difference between rural and urban-dwelling men in logistic regression analysis with rural-dwelling men having a 70 % higher risk of locally-advanced disease (OR = 1.70, 95 % CI: 1.03-2.79; *p* = 0.038).

**Conclusion:**

Rural-dwelling men presented with more advanced prostate cancer compared to urban-dwelling men. As both rural and urban-dwelling men are of predominant African descent it is likely that these differences are due to differences in access to care, screening practice and awareness of the disease.

## Background

Caribbean men with prostate cancer (PCa) have their disease diagnosed at a later stage and suffer a worse outcome compared to US born men of Afro-Caribbean descent [1]. Disparities in prostate cancer incidence and mortality have been attributed to different factors by a number of authors. In comparing outcomes of PCa treatment in African American (AAM) and Afro-Caribbean men (ACM) with Caucasian American men (CAM), being of African descent was found to be an independent predictor of a worse survival [2]. It was reported based on an analysis of the Surveillance, Epidemiology and End Results (SEER) database in the United States that only 29 % of the disparity in the incidence of metastatic PCa between AAM and CAM was explained by differences in PCa screening practice, comorbidities and income; and none of the disparity in loco-regional disease, suggesting biological factors related to race may be more important [3]. Others have found that factors primarily related to inequities in access to medical care such as distance to a urologist [4], physician recommended treatment-related disparities wherein Black men receive less aggressive stage and age inappropriate treatment [5], lack of insurance and low household income are some of the socially determined factors to be blamed [6]. It is likely that both biological factors related to African descent as well as social/environmental factors are important in explaining PCa disparities.

Jamaica, a country comprised primarily of persons of African descent, has high recorded PCa incidence and mortality rates for its metropolis, Kingston and St. Andrew (KSA). Age-standardised incidence and mortality rates have been reported at 78.1/100,000/year [7] and 53.9/100,000/year [8] respectively. The clinico-pathologic profiles of Jamaican men with PCa accessing private urologic care and care at one of two tertiary care hospitals in the KSA metropolis have been described but the distribution of their profiles according to rural or urban residence was not reported [9, 10]. A lack of information on the clinico-pathologic profiles of rural versus urban-dwelling Jamaican men with PCa therefore exists.

In Jamaica there is no formal national screening policy for PCa [11] and men often present with advanced and symptomatic disease [12]. In one series 80 % of Jamaican men had symptomatic disease at presentation [9]. The usual pathway to diagnosis of PCa for the majority of Jamaican men is through referral to a urologist at a public hospital or one in private practice after being first seen for symptomatic disease at a health centre or general practitioner. A median prostate-specific antigen (PSA) level of 30.7 ng/ml for incident cases of PCa in Jamaica was reported with almost a third of men having a PSA level greater than 100 ng/ml [10].

While there are no significant differences in the predominant African heritage of Jamaicans within rural and urban settings, major differences may exist in ready access to medical care and medical information. For example, specialized urological services are more available in urban settings with tertiary care facilities and access to internet-based sources of information is less in rural areas. If access and awareness are critical determinants in PCa presentation and prognosis, then significant differences would be expected in the clinico-pathologic profiles of rural versus urban men. We therefore compared the initial clinico-pathologic profiles and treatments of rural and urban-dwelling men with PCa seen in private health care settings at initial presentation.

## Methods

The medical records of all men, whether still living or deceased, diagnosed with PCa from two private urologists’ offices were reviewed. One urologist was based in the country, in the south-western part of the island, while the other was based in Kingston but saw patients on a fortnightly basis in the south-western region of Jamaica as well. The study was approved by the Ethics committee of the University of the West Indies.

Cases were men with histologically confirmed PCa or men with overwhelming clinical, biochemical and/or radiological evidence of PCa who did not undergo biopsy but were presumed to have the disease. The latter comprised less than 1 % of cases and invariably had PSA levels > 500 ng/ml.

Information on patients’ age, address, date at presentation, initial PSA level, local tumour stage based on digital rectal examination (DRE) findings, biopsy Gleason score and initial treatment received were extracted from the patients’ files. The records spanned a 12 year period from 1999 to 2011.

### Setting

Although urban is defined by the Statistical Institute of Jamaica (STATIN) as a place that “has a population of 2000 or more persons and provides a number of amenities and facilities which in Jamaica indicates modern living”, for the purpose of this study only cities and capitals of parishes were considered urban. The capital city of Jamaica, the largest urban centre, is located within the geographic boundaries governed by the municipal authority of the parishes of Kingston and St. Andrew (KSA) found in the south-eastern part of the island and has a population of 670,000 people. It has two tertiary-level care public hospitals and 12 urologists.

The designation ‘rural’ was applied to patients residing outside of named capital towns and cities in the south-western part of Jamaica which was one of the two study settings.

**Fig. 1:**
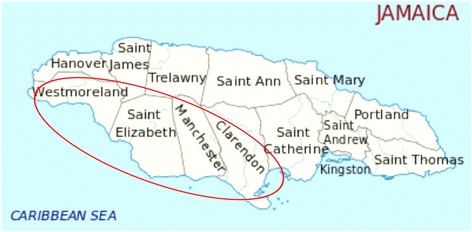
Map of Jamaica showing the southwestern region with a population of 738,292 served by one fixed urologist

One urologist practices solely in the south-western region of the island, a country area comprised of 4 parishes – Westmoreland, St. Elizabeth, Manchester and Clarendon with a combined population of 738,000 people (Fig. 1). He is the only fixed practicing urologist in this region. His practice is comprised of 3 locations, Junction (St. Elizabeth), Mandeville (Manchester) and May Pen (Clarendon). Mandeville and May Pen are the capital towns for their respective parishes of Manchester and Clarendon and are considered urban centres. Junction is a small non-capital town in the parish of St. Elizabeth and is considered rural for the purpose of this study.

The south-western region of the island consists of many rural villages and extremely remote areas with very hilly terrain. The majority of patients accessing private urological care for prostate cancer from this region of the island would be seen by the fixed country urologist.

A minority of country patients may be managed by general surgeons, opt to go to a city-based urologist in Kingston or Montego Bay, or may be seen by itinerant urologists who travel from Kingston to the country on an infrequent basis.

The city-based urologist practices in Kingston and May Pen, the latter being the capital town for the parish Clarendon and therefore an urban centre, situated in the mid-southern portion of the island (Fig. 1) where he sees patients fortnightly. Patients seen by both urologists in the country offices may be from either urban or rural settings. Patients from the country seen in Kingston by the Kingston-based urologist were excluded from the analysis.

### Clinical and pathologic assessments

Both urologists used the AJCC TNM staging system to assess local prostate tumour stage clinically on DRE. However, because of slight differences in the classification of the sub-categories of T2 based on the 1992 versus the 2003 TNM staging systems, only general T categories (T2, T3 and T4) and T1c were used in the analysis to minimise misclassification errors. PSA levels done in KSA were done by one of three medical laboratories while those outside of Kingston and St Andrew were done by a limited number of medical laboratories inclusive of the same three laboratories in Kingston which had branches in the country.

Most prostate biopsies done in KSA were done by a single radiologist under transrectal ultrasound guidance taking a minimum of 12 to 16 cores. Prostate biopsies outside of KSA were mostly done under transrectal ultrasound guidance but men with palpable, locally advanced tumours (T3 and T4) commonly had finger-guided biopsies, taking fewer cores in the process. Specimens from KSA were evaluated by a single pathologist in almost all cases and this was the same for outside of KSA.

### Analyses

The clinico-pathologic features of men with prostate cancer from urban and rural areas were described using summary tables. As appropriate, means of the two groups were compared using the Student’s t-test, median PSAs were compared with the Wilcoxon rank-sum test. Clinical T stage was re-categorised into 2 groups organ-confined (T1 and T2) and locally-advanced (T3 and T4) disease and associations with geographic location determined using Pearson’s chi squared test. P-values of <0.05 were deemed statistically significant.

Regression analysis was also done to control for confounding and identify independent predictor variables for rural–urban status. The variables included in the regression analysis were those associated with rural/urban location in bivariate analyses. The outcome ‘rural’ and ‘urban’ was coded as one and zero respectively. Potential independent variables identified through bivariate analysis were entered in the model. For logistic regression Gleason scores were recoded as 5–6 ‘low’, 7 ‘intermediate’ and 8–10 ‘high’. DRE stage was converted to a binary variable with Stages T1c and T2 being combined as ‘organ-confined disease’ and Stages T3 and T4 being ‘locally-advanced disease’ (i.e. disease extending beyond the prostatic capsule).

The Statistical Package for the Social Sciences (SPSS) version 17 was used for the analyses.

## Results

Three hundred and seventy-six (376) cases were reviewed. The majority (59.0 %) were from rural addresses of which 79.3 % were from the practice of the country urologist. There were significant differences in the origin of patients utilizing the urology practices (*p* < 0.001). Of those seen outside of KSA (298), approximately 25 % was urban-dwelling in contrast to that of the Kingston-based urologist where all were urban dwelling (Table 1).

**Table 1:** Patient characteristics [*n*, (%)]

Variable	Categories	Urban	Rural
*Respondents*	Total	154 (41.0 %)	222 (59.0 %)
*Practice*	Urologist 1	78 (100)	0 (0)
	Urologist 2	76 (25.5)	222 (74.5)
*Age Group*	<50	2 (1.1)	2 ( 0.9)
	50-59	29 (50.9)	28 (49.1)
	60-69	58 (46.5)	61 (54.5)
	70-79	53 (35.6)	96 (64.4)
	80-89	15 (36.1)	29 (65.9)
*DRE Stage*	T1	50 (53.3)	50 (46.2)
	T2a	28 (49.1)	29 (50.9)
	T2b	13 (36.1)	23 (63.9)
	T3	32 (34.8)	60 (65.2)
	T4	15 (26.3)	42 (73.7)

Data on age were available for 97.3 % (366) of the cases. Ages ranged from 45 to 88 years. The median age was 70 years (IQR =16). The modal age category was 70–79 years (40.7 %); almost 1 in 3 persons were in the 60–69 age group. Only 4 cases (1.1 %) were less than 50 years old.

When the data was disaggregated by rural–urban residence, there were statistically significant differences in age distribution (Wilcoxon Rank Sum test; *p* = 0.034). Rural men were typically older than urban men, the median ages being 72 (IQR = 13) and 68.5 (IQR = 13) years respectively. Similar findings were also noted by urological practice setting with the country urologist seeing older patients when compared with the urologist in the city; (median age: 72 years versus 65 years).

Documented levels of PSA varied from 0.9 to 1841 ng/ml, in a highly and positively skewed distribution. The overall median PSA level was 19.0 (IQR = 36.4). Rural-dwelling men typically had higher PSA levels at presentation than urban men. Median PSA levels for rural-dwelling men of 22.9 ng/ml (IQR =43.5) versus 18 ng/ml (IQR = 22.4) for urban-dwelling men were observed. This difference was statistically significant (Wilcoxon Rank Sum test; *p* = 0.001).

Approximately 30 % of men were classified on DRE examination as having stage T1c disease, and slightly more than a quarter of men as having T3 disease. 26.3 % of men had T2 disease (16.3 % T2a and 10 % T2b) and 16.3 % had stage T4 disease. By rural–urban distribution, there was a statistically significant difference with regard to local stage of disease at presentation. Disproportionately higher percentages of rural men presented with more locally-advanced disease (χ^2^ = 15.02, df = 4, *p* = 0.005). While rural men constituted 59 % of study participants, they comprised 65.2 % and 73.7 % of those with T3 and T4 disease respectively.

**Fig. 2:**
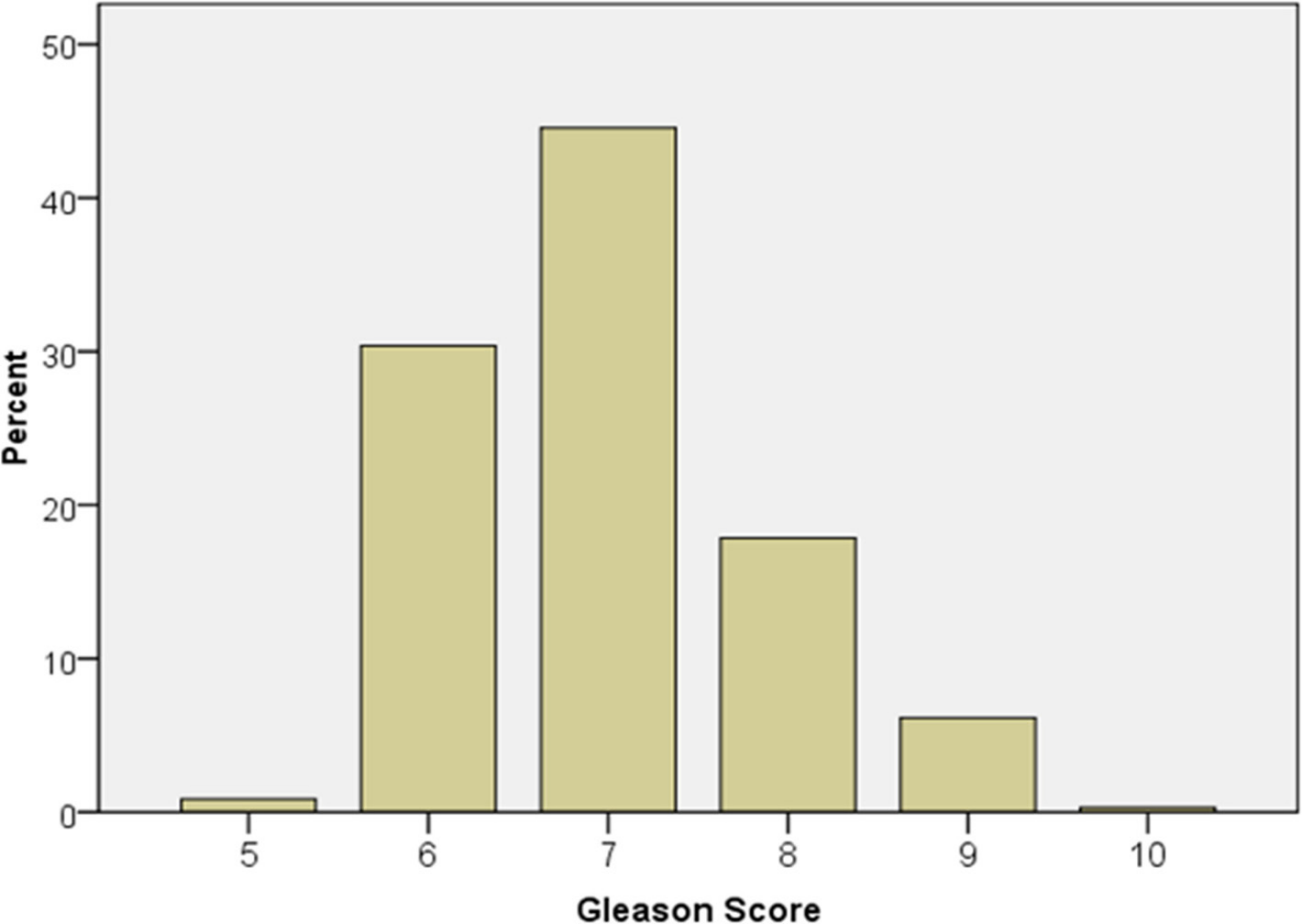
Distribution of Gleason Scores

Gleason scores ranged from 5–10, the overall mean score being 6.99 (sd =0.89) (Fig. 2). Three quarters of the cases (75 %) had scores in the 6–7 range, 17.8 % of cases had a Gleason score of 8, 6 % had Gleason scores of 9 and 0.3 % had Gleason scores of 10. High grade disease (Gleason score 8–10) accounted for 24.8 % of cases overall. Rural men had a statistically significant higher mean Gleason score than urban men (t =1.98, df =357, *p* = 0.048).

**Fig. 3:**
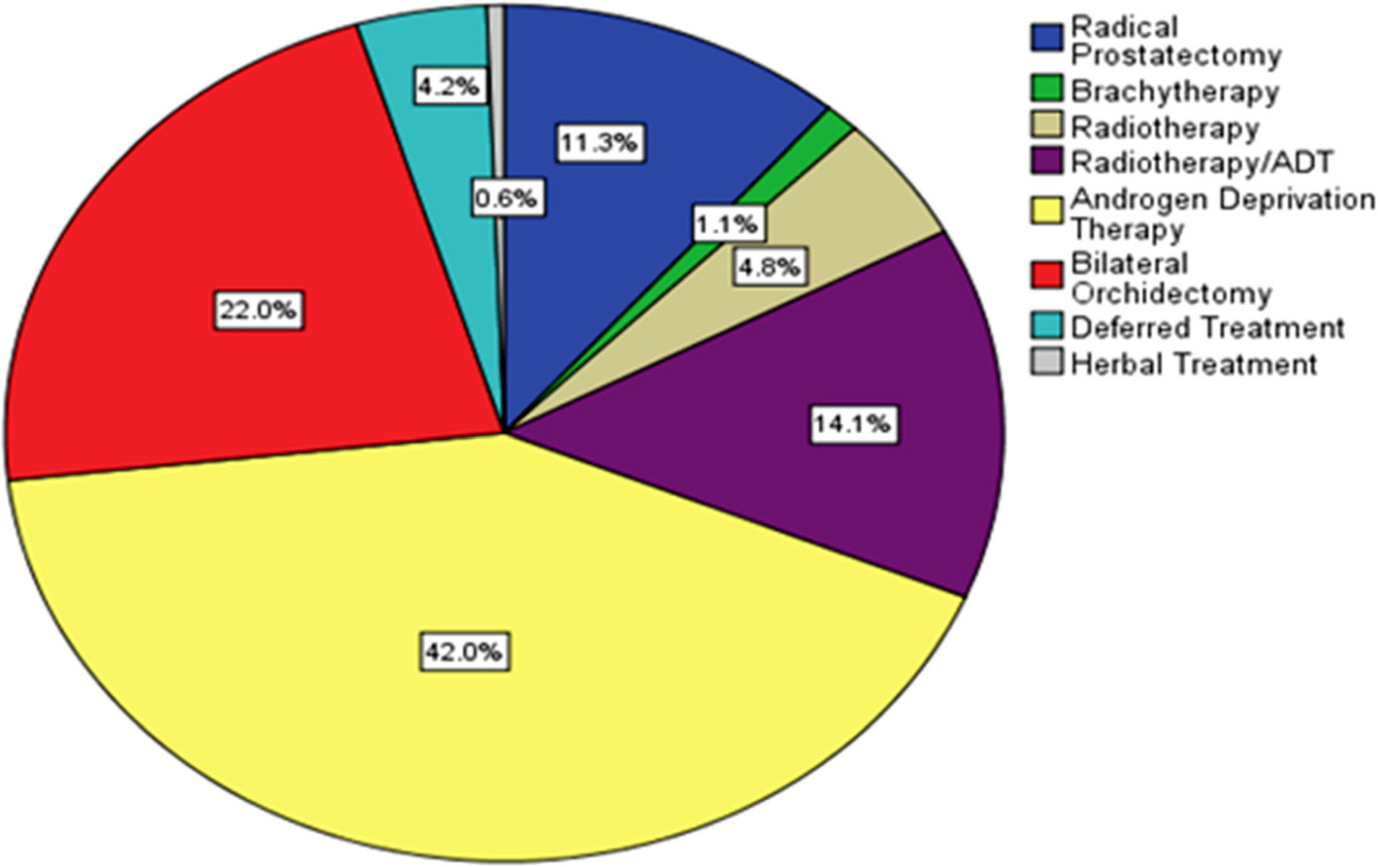
Initial Therapy at presentation

Figure 3 shows the distribution of the initial modes of therapy pursued with patients. Androgen deprivation therapy (ADT) (42 %) and bilateral orchidectomy (22 %) were the most frequently employed therapies. About 1 out of every 7 men received a combination of radiotherapy and ADT as the initial therapy for locally advanced disease. Watchful waiting and active surveillance (deferred treatment) as well as patient-initiated herbal treatment together totaled less than 5 % of initial treatment instituted. As initial therapy, significantly greater proportions of rural-dwelling patients (27.3 %) had bilateral orchidectomies than urban-dwelling patients (13.7 %) (χ^2^ = 9.2, df = 1, *p* = 0.002). In contrast, radical prostatectomy was more common among urban-dwelling (20.1 %) than rural dwelling men (5.6 %) Androgen deprivation therapy was equally used for urban and rural dwelling men as initial therapy (41.0 % and 42.6 % respectively).

By practice, the city-based urologist prescribed ADT using LHRH analogues (32.8 %) and performed radical prostatectomy (31.3 %) at initial therapy compared with the rural-based urologist where ADT using LHRH (44.0 %) and bilateral orchidectomy (24.4 %) were most frequently used.

In bivariate analyses, type of treatment was associated with area of residence and with DRE tumour stage. DRE tumour stage was also associated with area of residence. This pointed to the potential for confounding of the relationships observed. As type of treatment exhibited multicollinearity with DRE tumour stage, only the latter variable was used as a covariate in regression analysis.

A binary logistic regression model to identify independent predictors of rural/urban status was developed. Rural/urban status was the outcome. Variables identified as associated in bivariate analyses (PSA level, Age category, DRE stage and Gleason score category) were entered as predictors. Table 2 shows the results obtained.

**Table 2:** Predictors of geographic (rural–urban) residence among respondents

Variable	B	S.E.	*p*-value	Odds Ratios (95 % C.I.)
PSA level	.000	.001	.362	1.00(1.00-1.01)
DRE tumour stage category^a^ *(locally-advanced/organ-confined )*	.528	.254	..038	1.70(1.03-2.79)
AGE				
*<50 (reference category)*				
*50-59*	-.085	1.051	.936	0.92(0.12-7.21)
*60-69*	.082	1.035	.937	1.09(0.14-8.23)
*70-79*	.491	1.031	.634	1.63(0.22-12.23)
*80-89*	.721	1.075	.502	2.05(0.35-16.9)
Gleason Score Category				
* Low (reference category)*				
*Intermediate*	.251	.269	.350	1.29(0.76-2.18)
*High*	.521	.335	.120	1.68(0.87-3.25)

First category is the reference category for categorical covariates

All reference categories were coded as ‘0’

^a^indicates statistically significant predictors in logistic regression model with these variables

Dependent variable coded as Rural = 1, Urban = 0 (reference)

DRE stage was identified as the only significant independent predictor after controlling for age, Gleason score and PSA levels. Subjects with disease extending beyond the prostate (locally-advanced disease) were 1.7 times more likely to be rural persons than to be urban dwellers (OR = 1.7, 95 % CI = 1.03 - 2.29).

## Discussion

This study found that rural-dwelling men had a 70 % higher risk of having locally advanced disease when compared to urban-dwelling men based on DRE findings and this was clinically and statistically significant. It is likely that a lack of awareness of the disease, comparatively lower rural screening activity, diminished access to readily available and affordable general medical and specialist urologic care, and comparatively lower economic means contributed to the observed results.

Rural-dwelling patients had a significantly greater number of bilateral orchidectomies as initial treatment which is not only due to more advanced tumours but also reflect differences in urologists’ practice preferences as well as issues related to affordability and concerns with patient compliance. Similarly, differences in rates of radical prostatectomy was not simply a function of stage of disease, but was influenced by access and availability with it being more available in KSA. Hence, practice preferences rooted in the local realities related to cost, access, and compliance and limited follow-up are putative factors that help explain why orchidectomy may have been used for ADT in rural men with advanced disease and LHRH analogues was more frequently opted for by the city-based urologist.

When compared to the clinico-pathologic profiles of PCa in Jamaican men described by Shirley et al. [9] and Coard et al. [10] neither of whom differentiated between rural and urban dwelling in a clinical series of men diagnosed and treated in Kingston, median PSAs of 37 ng/ml and 30.7 ng/ml respectively were described which are higher than the overall median PSA of 19 ng/ml and the median rural PSA of 22.9 ng/ml in this study. These case series included patients diagnosed between 1993 to 1997, and 2000 to 2005 respectively. Secular trends of increased and more widespread use of PSA-based screening and greater awareness and understanding of the disease may account for these differences. Overall, 56 % of men in this study had localized disease (T1 and T2) on DRE compared to 63 % in Shirley’s study. Aiken et al. reported in an earlier study that 51 % of Jamaican men with newly diagnosed PCa had localized disease [12]. The distribution of Gleason scores in this study was similar to that reported by Coard. Shirley’s study had an inexplicably high percentage of high grade cancers which is most unusual.

Cadmium, a putative risk factor for PCa [13], is known to exceed the upper limits of safe levels in the soil in the parishes of Manchester and St. Elizabeth, a known bauxite belt [14, 15]. The concentration of cadmium found in yam tubers grown in these soils is as high as 55 % of that present in the soil and possibly 6.4 % is bioavailable on human consumption [16]. It is therefore possible that the differences seen in this study may be partly due to the cadmium levels in the soil. However, this is highly speculative and would require further study to verify an association locally.

Barriers to accessing urological care would have differentially affected the rural patients and may partly account for the differences observed. Geographic barriers exist due to the very hilly rural terrain, poorly maintained roads, and inconsistent transportation. Financial barriers to accessing urological care in the country due to less medical insurance coverage and absent or inconsistent income would also account for the differences noted. For example the health insurance coverage was 14 % in rural Jamaica versus 24 % in the Kingston Metropolitan Area at the midpoint of the period studied [17]. The single fixed country urologist covers the southwestern region of the island with a population of 738,000, whereas 12 urologists cover the KSA region which has a population of 670,000 and this contributes to the geographic maldistribution of access to urological care in the Island [11].

Study limitations included not having a record of socioeconomic indices such as educational attainment which may have confounded associations between location of residence and indices of prognosis. PSAs were measured by different laboratories, using different methods of assay, and this could have introduced increased variability in the PSA readings. This may contribute to a failure to detect an association between PSA levels and rural–urban residence in statistical analyses. Misclassification of tumour stage in local tumour stage assessment could arise due to differences in the interpretation of DRE findings; however this was minimized by combining the local DRE-based staging into 2 broad categories.

## Conclusion

Rural-dwelling men presented with more locally-advanced prostate cancer compared to urban-dwelling men. Since these men are generally of similar racial composition it is likely that these disparities are due to differences in access to care, screening practice and level of awareness of the disease. Efforts to reduce the morbidity and mortality from PCa in Jamaica must recognize these issues when public health programs and clinical services are developed for rural and urban clientele.

## Abbreviations

PSA: Prostate-specific antigen
AAM: African-American men
DRE: Digital rectal examination
CAM: Caucasian American men
PCa: Prostate cancer
ACM: Afro-Caribbean men
KSA: Kingston and Saint Andrew
LHRH: Luteinizing hormone releasing hormone
ADT: Androgen deprivation therapy
TNM: Tumor Nodes Metastases
STATIN: Statistical Institute of Jamaica
AJCC: American Joint Committee on Cancer

## References

[1] Mutetwa B, Taioli E, Attong-Rogers A, Layne P, Roach V, Ragin C (2010). Prostate cancer characteristics and survival in males of African Ancestry according to place of birth: data from Brooklyn-New York, Guyana, Tobago and Trinidad. Prostate.

[2] Ritch CR, Morrison BF, Hruby G, Coard KC, Mayhew R, Aiken W (2013). Pathological outcome and biochemical recurrence-free survival after radical prostatectomy in African-American, Afro-Caribbean (Jamaican) and Caucasian-American men: An international comparison. BJU Int.

[3] Taksler GB, Keating NL, Cutler DM (2012). Explaining racial differences in prostate cancer mortality. Cancer.

[4] Holmes JA, Carpenter WR, Wu Y, Hendrix LH, Peacock S, Massing M (2012). Impact of distance to a urologist on early diagnosis of prostate cancer among black and white patients. J Urol.

[5] Nambudiri VE, Landrum MB, Lamont EB, McNeil BJ, Bozeman SR, Freedland SJ (2012). Understanding variation in primary prostate cancer treatment within the Veterans Health Administration. Urology.

[6] Xiao H, Tan F, Goovaerts P (2011). Racial and geographic disparities in late-stage prostate cancer diagnosis in Florida. J Health Care Poor Underserved.

[7] Gibson TN, Hanchard B, Waugh DM N (2010). Age-Specific Incidence of Cancer in Kingston and St Andrew, Jamaica, 2003–2007. West Indian Med J.

[8] Blake G, Hanchard B, Mitchell K, Waugh N, Wolff ES C (2002). Jamaica Cancer Mortality Statistics, 1999. West Indian Med J.

[9] Shirley SE, Escoffery CT, Sargeant L a, Tulloch T (2002). Clinicopathological features of prostate cancer in Jamaican men. BJU Int.

[10] Coard KCM, Skeete DH (2009). A 6-year analysis of the clinicopathological profile of patients with prostate cancer at the University Hospital of the West Indies, Jamaica. BJU Int.

[11] Morrison BF, Aiken WD, Mayhew R (2014). Current state of prostate cancer treatment in Jamaica. ecancer.

[12] WD Aiken, T Tulloch, V Freeman, F Bennett, K Coard, B Panton, et al. Differences in patient characteristics in black men with prostate cancer from Jamaica and Chicago. In *Proc Am Soc Clin Oncol 22: 2003 (abstr 1764)*; 2003.

[13] Luevano J, Damodaran C (2014). A review of molecular events of cadmium-induced carcinogenesis. J Environ Pathol Toxicol Oncol.

[14] Lalor GC (2008). Review of cadmium transfers from soil to humans and its health effects and Jamaican environment. Sci Total Environ.

[15] Lalor GC, Raltray R, Simpson P, Vutchkov M (1998). Heavy Metals in Jamaica. Part3: The Distribution of Cadmium in Jamaican Soils. Rev Int Contam Ambient.

[16] Spence A, Hanson RE, Grant CN, Hoo Fung L, Rattray R (2014). Assessment of the bioavailability of cadmium in Jamaican soils. Environ Monit Assess.

[17] Jamaica Survey of Living Conditions 2006 [http://www.pioj.gov.jm/ResearchandData/SocialSectorsReports/tabid/131/Default.aspx]

